# Prognostic role of ABO blood group and Rhesus factor in cirrhotic patients with hepatocellular carcinoma

**DOI:** 10.1038/s41598-019-55685-8

**Published:** 2019-12-13

**Authors:** Alihan Oral, Tolga Sahin

**Affiliations:** 1 Department of Intenal Medicine, Faculty of Medicine, Demiroglu Bilim University, 34360 Istanbul, Turkey; 2 Department of Gastroenterology, Faculty of Medicine, Demiroglu Bilim University, 34360 Istanbul, Turkey

**Keywords:** Cancer, Biogeochemistry, Gastroenterology

## Abstract

Hepatocellular carcinoma (HCC) is one of the most common types of cancer worldwide. There are many factors in the etiology of HCC such as hepatitis B virus (HBV), hepatitis C virus (HCV), alcohol, obesity, smoking and aflatoxin. Many types of cancer are assumed to be associated with ABO blood group and Rhesus factor (RH). In this study we aimed to evaluate the relationship between tumor characteristics and overall survival (OS), ABO blood group and RH factor in patients with HCC. A total of 507 patients with chronic liver disease (252 patients with HCC and 255 patients without HCC) were included in the study. All demographic, clinic and laboratory (biochemical parameters and blood type) features were collected retrospectively. The mean age of the patients was 54.50 ± 9.30. There was no significant difference in both ABO groups and RH factors between the two groups. We found that vascular invasion rate of the tumor was higher in the B blood group and multicentric localization of tumor was significantly higer in patients with positive RH but there was no difference between OS in ABO and RH blood groups. In addition, the tumor was less multicentric in the AB blood group. Blood groups and RH factor can be used to predict the prognosis in cirrhotic patients with HCC.

## Introduction

Hepatocellular carcinoma (HCC) is one of the most common types of cancer worldwide^1^. It is also one of the leading causes of cancer-related deaths^2^. There are many factors in the etiology of HCC such as HBV, HCV, alcohol, obesity, smoking and aflatoxin^3,4^. Moreover, many factors have been found to be predictive for HCC such as advanced age, high alpha feto protein (AFP) level and presence of cirrhosis^5^.

Nowadays, many types of cancer are assumed to be associated with ABO blood group and Rhesus factor (RH). Pancreatic, gastric, skin and ovarian cancers were found to be associated with ABO blood group and RH factor in different studies^6–9^. Although there are some studies claim relationship between HCC and blood groups, the issue is still unclear^10–12^. In addition, there are only a few studies in the literature about whether there is a relationship between the characteristics of cancer and ABO blood type in patients with HCC^13^. Therefore, we aimed to evaluate the relationship between tumor characteristics and overall survival (OS), ABO blood group and RH factor in patients with HCC in this study.

## Results

**Figure 1:**
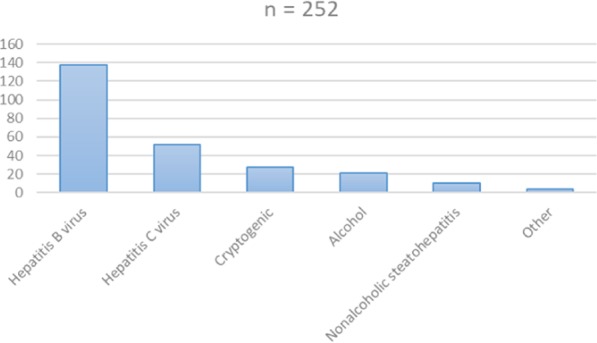
Etiology of hepatocelluler cancer.

Two hundred and fifty two cirrhotic patients with HCC and two hundred and fifty five cirrhotic patients without HCC were included in the study. The mean age of the patients was 54.50 ± 9.30 and 52.9% were male, 47.1% were female. Age, BMI and alanin aminotransferase (ALT) values were similar in HCC and non-HCC cirrhotic groups. AFP, albumin values and male gender were significantly higher in HCC group rather than non-HCC group. Aspartate aminotransferase (AST), total biluribin and international normalized ratio (INR) values and male gender were significantly lower in HCC group rather than non-HCC group. Hepatitis B virus was the most common etiologic factor in HCC group. (Table 1, Fig. 1).

**Table 1:** Demographic and laboratory findings and AFP value of study population.

	HCC (n = 252)	Non-HCC (n = 255)	P
Age	56,38 ± 9,31	52,30 ± 9,21	0,054
Gender (Female/Male, %)	42,4/57,6	52,9/47,1	0,013
Body mass index (kg/m^2^)	27,37 ± 3,83	27,86 ± 4,76	0,413
ALT (IU/L)	58,83 ± 4,07	56,88 ± 53,44	0,122
AST (IU/L)	75,14 ± 55,20	97,26 ± 223,85	0,04
Total Biluribin (mg/dl)	2,53 ± 3,17	5,11 ± 6,94	<0,001
Albumin (g/dl)	3,42 ± 0,70	2,89 ± 0,57	<0,001
INR	1,46 ± 0,98	2,89 ± 0,56	<0,001
AFP (ng/mL)	159,98 ± 877,26	13,27 ± 35,76	<0,001
Child–Pugh score	7,05 ± 2,13	9,30 ± 2,20	<0,001

ALT: Alanine aminotransferase; AST: Aspartat aminotransferase; INR: International normalization ratio; AFP: Alpha fetoprotein.

When the HCC and non-HCC groups were compared, there was no significant difference in both ABO blood and RH factor groups. The ratio of RH and ABO blood groups in HCC and Non-HCC groups are shown in Table 2. The characteristics of the tumor were evaluated in HCC group according to the ABO blood and RH factor. We found that the rate of vascular invasion was higher in the B blood group. In addition we also found that the rate of multicentric localization was significantly higher in positive RH factor group. Moreover the tumor was less multicentric in the AB blood group (Table 3).

**Table 2:** Comparison of study patients according to ABO blood group and Rh factor.

	HCC n (%)	non-HCC n (%)	P
O	72 (28,6)	84 (32,9)	0,286
A	114 (45,2)	100 (39,2)	0,170
B	44 (17,5	51 (20,0)	0,464
AB	22 (8,7)	20 (7,8)	0,717
Rh -	29 (11,5)	29 (11,4)	0,962
Rh +	223 (88,5)	226 (88,6)	0,982

**Table 3:** Relationship between the features of HCC and blood groups.

	Max. Tumor diameter (cm)	P	Total tumor diameter (cm)	P	Number of tumors	P
O	3,43 ± 2,24	0,264	5,27 ± 3,94	0,674	2,92 ± 2,79	0,218
A	3,57 ± 2,06	0,612	5,40 ± 3,71	0,658	2,71 ± 3,0	0,525
B	3,41 ± 1,61	0,635	4,76 ± 3,23	0,851	2,47 ± 2,35	0,319
AB	2,96 ± 1,04	0,693	3,22 ± 1,04	0,642	2,28 ± 3,52	0,351
RH +	3,31 ± 1,77	0,745	5,22 ± 3,68	0,077	2,58 ± 2,87	0,012**

*O blood group (or A, B and AB**)** and non-O group (both A, B, AB) were compared.

**P < 0,05.

When the patients were evaluated according to the maximum or total tumor diameter, no significant difference was found between ABO and RH groups. However, the number of tumors was significantly higher in RH positive patients (p: 0.012; Table 4).

**Table 4:** Comparison of HCC features according to ABO blood group and Rh factor.

	Vascular invasion n (%)		P	Multicentric HCC n (%)		P
	Yes	No		Yes	No	
O	12 (24,0)	60 (29,7)	0,424	36 (32,1)	36 (25,7)	0,262
A	20 (40,0)	94 (46,5)	0,406	56 (50,0)	58 (50,9)	0,174
B	14 (28,0)	30 (14,9)	0,028**	15 (13,4)	29 (20,7)	0,128
AB	4 (8,0)	18 (8.9)	0,838	5 (5,4)	17 (12,1)	0,032**
RH +	44 (88,6)	179 (88,0)	0,903	104 (%92,9)	119 (85,0)	0,047**

*O blood group (or A, B and AB**)** and non-0 group (both A, B, AB) were compared.

**P < 0,05.

**Figure 2:**
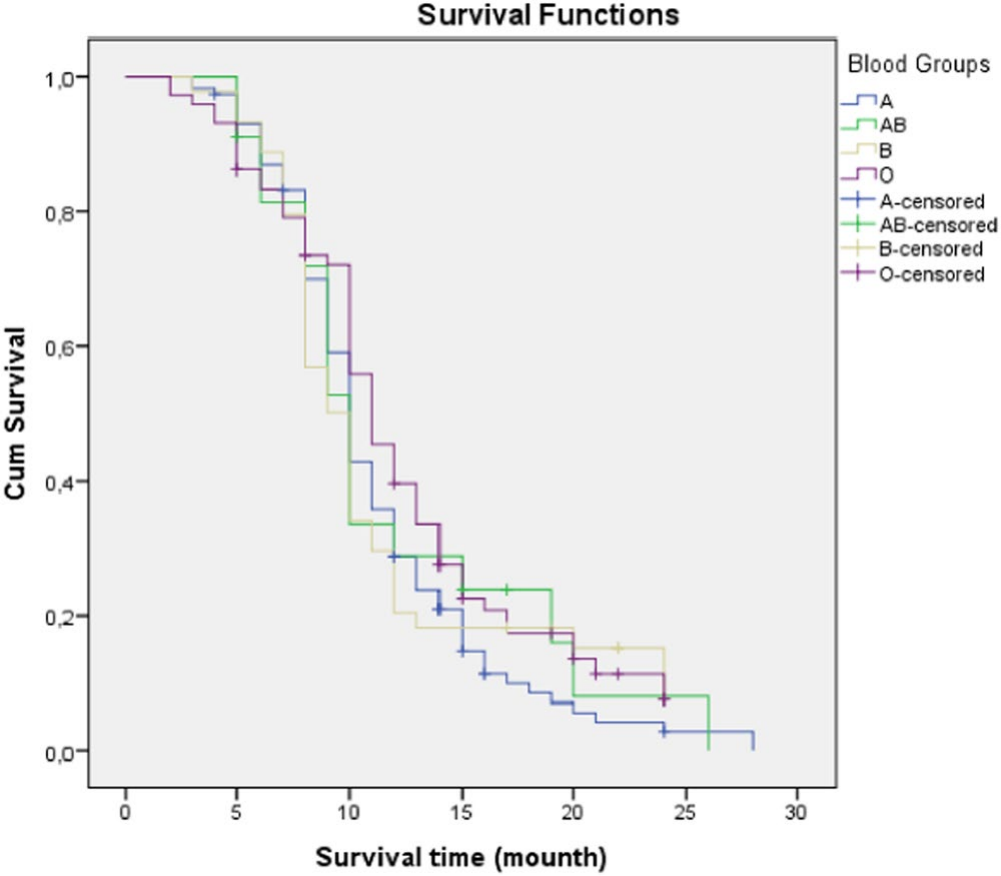
Survival analysis according to blood groups (ABO).

**Figure 3:**
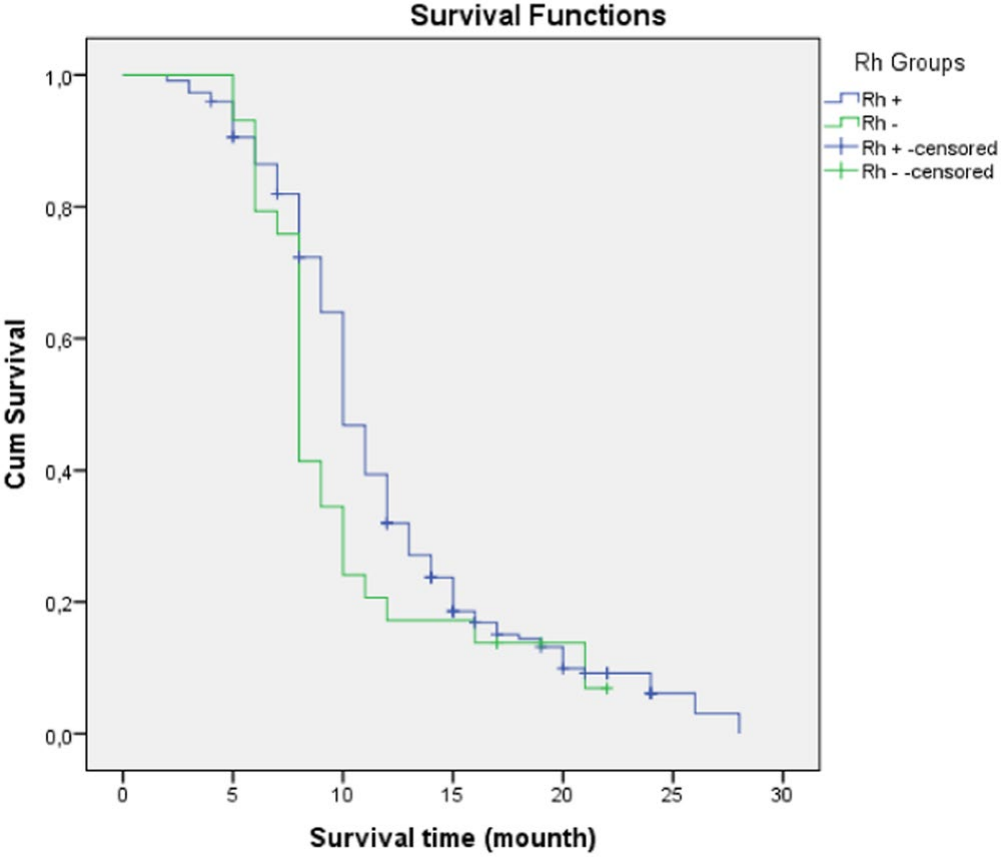
Survival analysis according to RH groups.

In OS analysis of HCC group; the median OS 11 months (95% CI: 10.9–12.4); the 6-, 12-, and 24-months OS rates were 85.6%, 39.8%, and 9.6%, respectively. There was no statistically significant difference in the effect of ABO and RH blood groups on OS (p: 0.491; p: 0.092, respectively) (Figs. 2 and 3).

## Discussion

In this study, there was no significant difference between HCC and non-HCC cirrhotic patients in terms of ABO blood group and Rh factor. There was no statistically significant difference in the effect of ABO and RH blood groups on OS of HCC. However, vascular invasion rate was higher in B blood group patients and the presence of multicentric localization of HCC was higher in RH positive patients. Interestingly in patients with AB blood group, HCC was less multicentric.

HCC is an aggressive tumor with a poor prognosis and its the third most common cause of cancer-related deaths worldwide^14^. In etiology, viral infections are at the forefront and the most common cause is hepatitis B virus^15^. AFP is the most commonly used serum tumor marker in routine practice in the diagnosis and screening of HCC. Many studies have shown that advanced age, male gender and high AFP level are predictive factors for HCC in patients with cirrhosis^5,16^. In our study, male gender and high AFP levels were found to be high in patients with HCC in compatible with the literature.

Obesity is known to be associated with liver cancer. In a US study, BMI > 35 was found to increase the risk of HCC development 4-fold compared to normal BMI^17^. In our study, although the BMI was higher in patients with HCC, the mean BMI was not at the level of obesity. Moreover, BMI values were similar with control group.

Data on the relationships between HCC and blood groups are still controversial. There are a limited number of clinical studies evaluating the relationships between HCC, ABO blood groups and RH factor in the literature^10–12,18,19^. Blood group A was found to be a risk factor for HCC development in some studies^11,12^. O blood group has been found to be low risk for HCC in many studies, but on the contrary, there are also studies showing that O blood group is high risk for HCC development^20^. Many studies have shown relationship between RH positivity and breast cancer^21,22^, but there are also studies showing no correlation between HCC and RH factor in literature^10^. There was no difference between HCC and non-HCC group patients in terms of ABO blood group or RH factor in our study.

Tumor necrosis factor-α (TNF-α) and epidermal growth factor gene have been revealed to be related with HCC risk^23,24^. ABO blood group antigen may be the source of systemic inflammation. TNF- α and soluble intercellular adhesion molecule 1 (sICAM-1) that are inflammation markers are associated with polymorphisms at the ABO locus^25,26^. Elevated levels of TNF-α play a role in the pathogenesis of liver inflammation and cancer pathogenesis and increased plasma levels of sICAM-1 have been suggested to be a predictor of HCC development. These markers may also be a predictive markers of HCC development and survival^11,27^. These factors can be considered as possible molecular mechanisms that explain the relationship between ABO blood group and HCC risk. However, the direct relationship between tumor characteristics and ABO and RH blood groups is still unclear. Several studies have found a relationship between ABO blood groups and RH with various types of cancers and their tumor characteristics^13,21,22^. Recently, in a study on breast cancer, a relationship was found between O blood group, tumor size, RH factor and estrogen receptor status^22^. In another study that was published recently, Rh factor was found to be associated with invasive tumor^21^. A large study from China, the author was found no correlation between tumor size of HCC, ABO blood groups and RH factor^13^. In that study the authors did not evaluated the vascular invasion and multicentric localization of tumor. The strength of our study is that many tumor features such as tumor size, number of tumors, multicentric localization and vascular invasion were investigated. In this respect, our study is one of the rare studies in the literature on this issue.

HCC is a very rapidly progressing malignant tumor^14^. In a recent study, the mean OS was 9 months. In the same study; HCC patients showed 1- and 2-year survival rates equal to 49.3% and 35.3%^28^. There are no enough studies showing the relationship between HCC survival and blood groups. In one study, AB and non-O blood group and in another study B and AB group were shown to have poor OS^13,29^. In this study, HCC patients showed 1- and 2-year survival rates equal to 39.8%, and 9.6% and there was no statistically significant difference in the effect of ABO and RH blood groups on OS. This may be due to the number of HCC patients were relatively small.

In our study, while aspartate aminotransferase (AST), total biluribin and international normalized ratio (INR) values were significantly lower in HCC group rather than non-HCC group, albumin value was significantly higher in HCC group rather than non-HCC group. This result may be due to the fact that the non-HCC group consists mostly of decompensated cirrhosis patients compared to the HCC patient group.

There are also some limiting factors in our study. First, our study was a single center study with retrospective design. Second, subgroups (KELL/lewis, Lutheran/P, Duffy/KIDD etc.) of blood groups were not evaluated. Third, patient groups were composed of single ethnic origin and number of HCC patients were relatively small.

## Conclusion

Relationships between HCC and blood groups are still unclear and there is not sufficient data on this issue in the literature. In our study there was no difference between HCC and non-HCC group patients in terms of ABO blood group and RH factor. However vascular invasion and multicentric localization of tumor was significantly related with blood group and RH factor. Vascular invasion was higher in the B blood group and multicentric localization of tumor was higher in positive RH factor group. There was no statistically significant difference in the effect of ABO and RH blood groups on OS of HCC. Despite the limiting factors, our study can make important contributions to the literature. More extended data can be obtained through new studies.

## Methods

Five hundred and seven patients over the age of 18 who were followed up with chronic liver disease in the gastroenterology outpatient clinic between January 2010–2019, were included in the study. Demographic (age, sex, height, weight, body mass index (BMI)), laboratory (biochemical tests, lpha fetoprotein (AFP) and blood type), Child-Pugh scores, etiology of HCC and imaging (ultrasonography, computed tomography, magnetic resonance and positron emission tomography) findings of the cases were retrospectively reviewed from the hospital information system and files. Twenty four months survival of the patients was evaluated. Several milliliters used fresh blood on ethylene diamine tetracetic acid (EDTA) as a preservative for testing blood group antigens by agglutination technique for ABO and Rh blood groups. The patients were divided into two groups as HCC and Non-HCC cirrhotic group. All patients had histopathologically diagnosed HCC with liver biopsy. Other features of the tumor (number, size, etc.) were determined by radiological imaging methods. Patients under 18 years of age and whose blood type data could not be obtained were excluded. This study was conducted according to the guidelines laid down in the Declaration of Helsinki and all procedures involving human subjects were approved by the Demiroğlu Science University Ethics Committee (approval number 2019-16-04; approved on 08.06.2019) and informed consent was obtained from all participants.

Statistical analysis; numerical data were given as mean-standard deviation. Data with normal distribution were calculated with Student-t test and data without normal distribution were calculated using Mann-Whitney U test. Categorical data were calculated using Chi-square test. OS was calculated from the date of diagnosis to the date of death or the last follow-up. OS was estimated using the Kaplan–Meier method, and the statistical significance of differences between curves was tested using the log-rank test. Statistical analysis was performed using SPSS 21.00 programme and the confidence interval (CI) was accepted as 95%. P < 0.05 was accepted as significant statistical result.
